# Supplementary figures and images for: Correction of Italian under-reporting in the first COVID-19 wave via age-specific deconvolution of hospital admissions
Source: PLoS One. 2023 Dec 7;18(12):e0295079. doi: 10.1371/journal.pone.0295079 (PMC10703316; doi:10.1371/journal.pone.0295079)

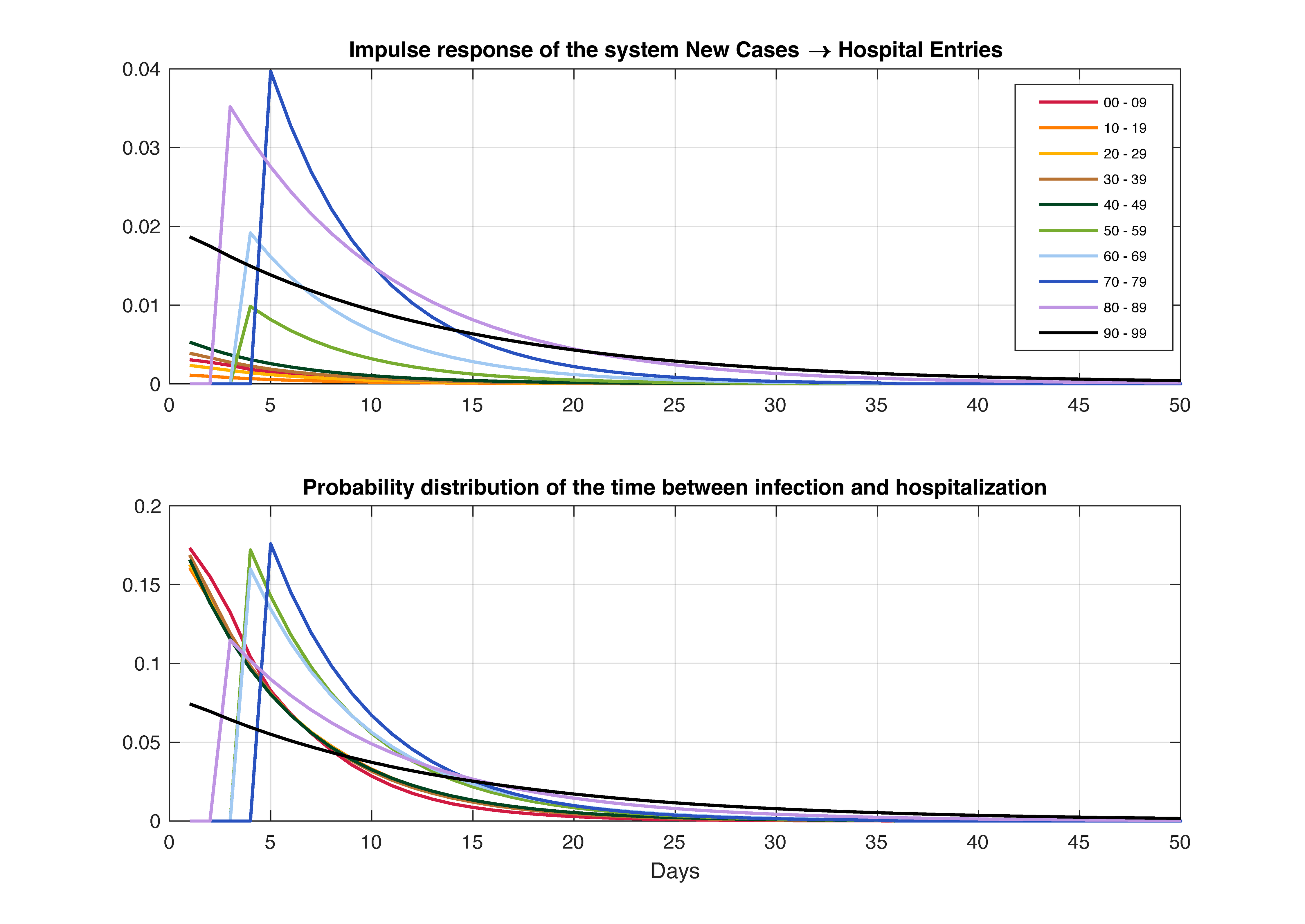

Supplement: S1 Fig — The upper panel shows the ten different impulse responses. The lower panel of the figure shows ten different probability distributions of elapsed time from positivity. Among these distributions, the age groups up to 49 years old share a similar pattern, with a shorter mean compared to the three age groups between 50 and 79 years old. The last two age groups exhibit the slowest decay rates. (TIF) [file pone.0295079.s001.tif]

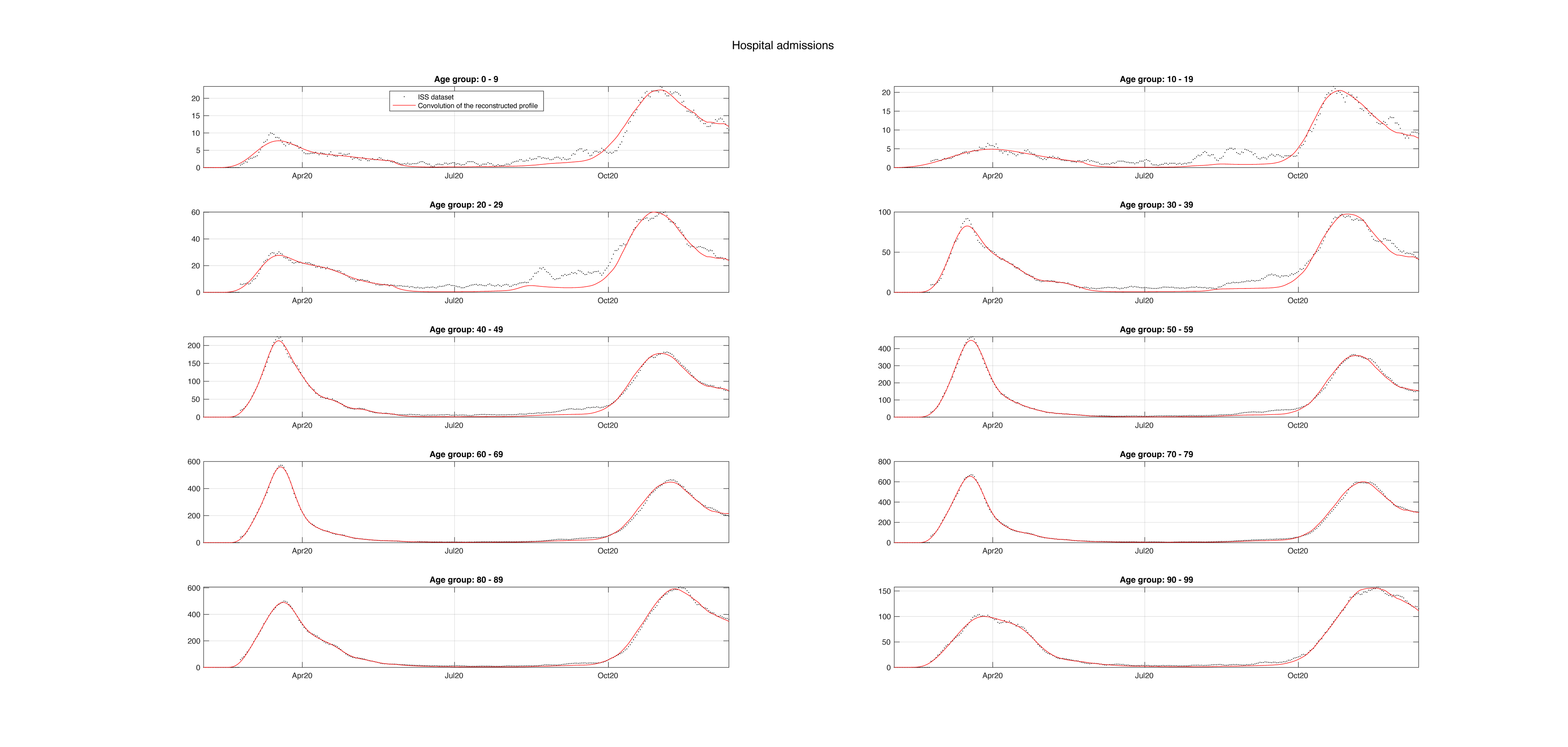

Supplement: S2 Fig — The hospital entry predictions for all age groups are presented. The model fits well, except for the younger age groups that, however, account for a smaller proportion of hospitalizations. Conversely, the fit for older age groups is satisfactory during the whole period. (TIF) [file pone.0295079.s002.tif]
