# Supplementary material for: Correction of Italian under-reporting in the first COVID-19 wave via age-specific deconvolution of hospital admissions
Source: PLoS One. 2023 Dec 7;18(12):e0295079. doi: 10.1371/journal.pone.0295079 (PMC10703316; doi:10.1371/journal.pone.0295079)
Supplement: Attachment — Submitted filename: Response to Reviewers.pdf [file pone.0295079.s003.pdf]

November 3, 2023

Dear Professor Fabiana Zama,

We are pleased to reply to Rewiers' comments on Manuscript *Correction of Italian under-reporting in the first COVID-19 wave via age-specific deconvolution of hospital admission* [PONE-D-23-14782] - [EMID:7974616fa2338bdf].

We thank the anonymous reviewers for their constructive comments and valuable suggestions that helped us to improve the manuscript. The paper has undergone a careful revision in order to address all the issues raised by the reviewers, as detailed below in our point by point response to the comments. In the manuscript, all the changes have been indicated by using a yellow highlighter and a red text. Moreover, we complied with the format for revised manuscripts in our submission guidelines. Finally, we further explained the rationale for having to correct unreported cases in 2020.

Reply to the editor

(i) *Please ensure that your manuscript meets PLOS ONE's style requirements, including those for file naming.*
We used the journal template for LaTeX, hence the style requirements were met. Also, file names have been updated as requested.

(ii) *We note that you have included the phrase "data not shown" in your manuscript. Unfortunately, this does not meet our data sharing requirements. [. . .] if the data are not a core part of the research being presented in your study, we ask that you remove the phrase that refers to these data.*
Since the data is not relevant, we decided to remove the sentence from the paper.

(iii) *We notice that your supplementary figures are uploaded with the file type 'Figure'. Please amend the file type to 'Supporting Information'.*
We renamed the files as required.

(iv) *If you would like to make changes to your financial disclosure, please include your updated statement in your cover letter.* In the financial statement we would like to add the support the Italian Ministry for Research within PRIN 2022 program. The updated statement is: "This research was supported by EU funding within the NextGenerationEU-MUR PNRR Extended Partnership initiative on Emerging Infectious Diseases (Project no. PE00000007, INF-ACT), and the Italian Ministry for Research in the framework of the 2022 PRIN, Grant no. 2022LP77J4, Proliferation, Resistance and Infection Dynamics in Epidemics (PRIDE)."

Reply to the reviewer #1

(i) *Lines 27-41: an equation would help discuss this method, and facilitate the discussion.*
The following additional sentence was inserted: "More precisely, letting $u(t)$ denote new cases at time t, the delay adjusted model predicts deaths at time

t as $d(t) = \text{CFR}u(t - T)$, where T is the average delay between case recording and deaths."

(ii) *Line 99: explain what the function oe.m does (what method/algorithm implements)*
We completed the explanation in the following way: "The MATLAB function `oe.m` relies on prediction error minimization carried out by means of a Levenberg-Marquardt nonlinear least squares algorithm [5]".

(iii) *After line 140: why is it convenient to move to a linear programming framework, and why is it a good approximation? Also, it is not clear when do you actually use the linear programming approach.*
We agree with the reviewer. The lines were removed because they do not contribute significantly to the paper and shift attention to minor details.

(iv) *Line 274: it might be a terminology issue, but I would not classify the methods described in the paper as machine learning. Please clarify what methods do you refer to as machine learning.*
Thank you for the accurate comment. Since the term could generate a misconception, we preferred to remove "machine learning" from the text.

(v) *Line 281: Missing references (REFS).*
The references were successfully added: [4] and [6].

(vi) *Line 358: Missing reference [?].*
The reference has been restored: [3].

Reply to the reviewer #2

(i) *In the abstract, 'it is possible to computed corrected time series. . . ' should read 'it is possible to compute'.*
Thank you for pointing out the error. We fixed it.

(ii) *Line 15: 'published on [18]' should read 'published by ISTAT [18]'.*
Corrected. Thank you.

(iii) *Line 358: There is s a missing citation; after Pandemic Planning Scenarios.*
The reference has been restored: [3].

(iv) *I guess I missed the point; but the rationale of having to correct under-reported cases in 2020 should be explained. Is it that the method can now be used to make better predictions on the number of reported cases for an epidemic.*
Beside the paragraph in the introduction, we added another paragraph in the conclusion that summarizes the paper motivation:

Paragraph already present in the Introduction: The availability of a rigorous reconstruction of the first phase of the pandemic is valuable under several respects. In particular, we notice that some retrospective analysis were conducted by using official and underestimated data, e.g., [7], which makes problematic the assessment of public health interventions, including lockdowns and vaccination campaigns. For instance, without knowing the fraction of susceptible subjects it is impossible to run counterfactual simulations relative to NPIs and vaccination deployment. Our correction method is believed to prove useful also in connection with studies of the early and cryptic transmission [2] and those employing mobility data to study the spatial spread [1]: in both cases the reliability of the results would be compromised if heavily underreported cases were input in the

models.

New paragraph in the Conclusion: Finally, we highlight that correcting the underestimation of Italian cases during the first wave is valuable in several ways. According to the recorded data, Italy was one of the countries with the highest mortality rate, a fact that has prompted commentary and interpretation on the political and health management of epidemics. The availability of a more realistic estimate of actual cases is crucial to arrive at a fair assessment of the Italian response to the epidemic. Another point is the possibility of estimating the number of susceptible individuals at the beginning of the vaccination campaign. Again, without a reliable estimate, it is difficult or even impossible to quantify the impact of vaccines on overall mortality.

(v) *Some of the figures are rather unclear.* We had uploaded high resolution EPS figures, and positions of the legends are accurate. We suppose it could be a problem of the EPS viewer that possibly messed up. Of course, if there is really a problem with our graphics, we are available to solve the problem.

Thank you for your time in considering our manuscript and writing these insightful reviews.

Yours faithfully,

Simone Milanesi
Università degli Studi di Pavia, Dipartimento di Matematica "F. Casorati", Italy

Giuseppe De Nicolao
Università degli Studi di Pavia, Dipartimento di Ingegneria Industriale e dell'Informazione, Italy

References

[1] Duygu Balcan, Bruno Gonçalves, Hao Hu, José J Ramasco, Vittoria Colizza, and Alessandro Vespignani. Modeling the spatial spread of infectious diseases: The global epidemic and mobility computational model. *Journal of computational science*, 1(3):132–145, 2010.

[2] Simon Cauchemez and Paolo Bosetti. A reconstruction of early cryptic covid spread, 2021.

[3] CDC. COVID-19 Pandemic Planning Scenarios. `https://www.cdc.gov/coronavirus/2019-ncov/hcp/planning-scenarios.html`.

[4] Giulia Giordano, Franco Blanchini, Raffaele Bruno, Patrizio Colaneri, Alessandro Di Filippo, Angela Di Matteo, and Marta Colaneri. Modelling the covid-19

epidemic and implementation of population-wide interventions in italy. *Nature medicine*, 26(6):855–860, 2020.

[5] Lennart Ljung. System identification. In *Signal analysis and prediction*, pages 163–173. Springer, 1998.

[6] Istituto nazionale di statistica (ISTAT). Tavole di dati, id: 25653, tavola 1 (12 aprile 2021). `https://www.istat.it/it/archivio/256536`.

[7] Giovanni Sebastiani, Marco Massa, and Elio Riboli. Covid-19 epidemic in italy: evolution, projections and impact of government measures. *European journal of epidemiology*, 35:341–345, 2020.
